# Solar-Driven Freshwater Generation from Seawater and Atmospheric Moisture Enabled by a Hydrophilic Photothermal Foam

**DOI:** 10.1021/acsami.9b20291

**Published:** 2020-02-14

**Authors:** Siew-Leng Loo, Lía Vásquez, Uttam C. Paul, Laura Campagnolo, Athanassia Athanassiou, Despina Fragouli

**Affiliations:** † Smart Materials, Istituto Italiano di Tecnologia, Via Morego 30, Genova 16163, Italy; ‡ Dipartimento di Chimica e Chimica Industriale (DCCI), Università degli Studi di Genova, Via Dodecaneso 31, Genoa 16146, Italy

**Keywords:** interfacial solar evaporation, desalination, atmospheric-water harvesting, point of use, solar−thermal conversion

## Abstract

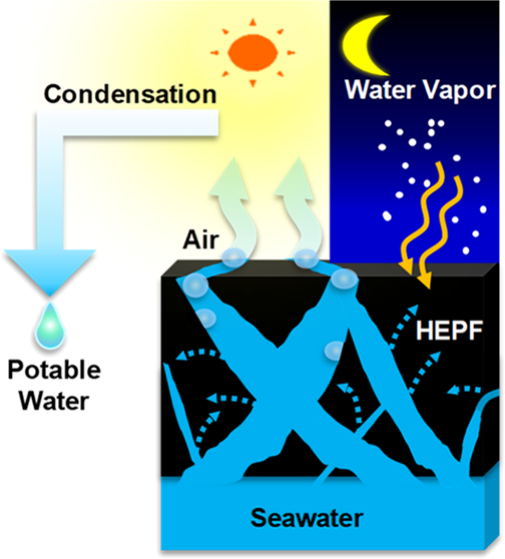

The accelerated increase in freshwater demand, particularly among populations displaced in remote locations where conventional water sources and the infrastructure required to produce potable water may be completely absent, highlights the urgent need in creating additional freshwater supply from untapped alternative sources via energy-efficient solutions. Herein, we present a hydrophilic and self-floating photothermal foam that can generate potable water from seawater and atmospheric moisture via solar-driven evaporation at its interface. Specifically, the foam shows an excellent solar-evaporation rate of 1.89 kg m^–2^ h^–1^ with a solar-to-vapor conversion efficiency of 92.7% under 1-Sun illumination. The collected water is shown to be suitable for potable use because when synthetic seawater samples (3.5 wt %) are used, the foam is able to cause at least 99.99% of salinity reduction. The foam can also be repeatedly used in multiple hydration–dehydration cycles, consisting of moisture absorption or water collection, followed by solar-driven evaporation; in each cycle, 1 g of the foam can harvest 250–1770 mg of water. To the best of our knowledge, this is the first report of a material that integrates all the desirable properties for solar evaporation, water collection, and atmospheric-water harvesting. The lightweight and versatility of the foam suggest that the developed foams can be a potent solution for water efficiency, especially for off-grid situations.

## 1 Introduction

The freshwater demand, driven by rapid population growth, climate change, industrialization and contamination of existing freshwater resources, is projected to increase at an even faster pace than the demands for energy.^1−3^ More alarmingly, the world has been projected to face a global water deficit, as an additional amount of about 2000 billion m^3^ of freshwater supply will be required by 2030 to meet the global demand.^4^ Currently, it has been estimated that about 2.1 billion people still lack access to a safe and readily available water supply, leading to an annual mortality of 361,000 children under the age of five.^5^ Addressing the issue of water supply is especially urgent for populations in remote locations, particularly those in poverty-stricken regions and in the aftermath of disasters, where conventional water sources and the required infrastructure may be absent.^6^ Therefore, low-cost, robust, and infrastructure-independent technologies with simple operation that can produce potable water at the “point of use” are urgently needed.

An attractive and viable platform to address this issue is the solar–vapor generators that use natural sunlight to produce potable water from unconventional sources, such as seawater and sewage, through solar-driven evaporation at their interfaces.^7−9^ For such a scope, tremendous advancements have been made in the development of photothermal materials, ranging from plasmonic nanoparticles,^10−13^ metal–organic frameworks,^14^ graphene/graphite,^15−17^ carbon nanotubes,^18,19^ carbonized natural products,^20−23^ and polymers,^24−30^ which are able to absorb light and efficiently transform it into heat. In addition, multifunctional solar–vapor generators that can adsorb and photodegrade water pollutants that further enhance their versatility in treating a wide range of contaminated wastewaters have been developed.^31−38^ In all cases, to achieve high evaporation efficiencies, a rational design of platforms that combines an efficient solar–thermal energy conversion, thermal insulation for heat localization, and a water-wicking mechanism for continual water supply is required.^8,9,39,40^ For this scope, prior studies have focused on the addition of external heat-insulation and water-wicking layers to the main structures of the solar–vapor generators.^13,24,31,41,42^ Although good performance can be achieved from such a strategy, potential issues arising from such a layered design strategy include the following: (i) interfacial incompatibility as it involves interfacing of bulk materials that may have completely different surface energies and (ii) complicated setups that limit their practicality.

Here, we report a hydrophilic and self-floating photothermal foam that shows high-rate evaporation without additional components, enabling an elegant and simple approach for water harvesting through solar evaporation. The foam, hereafter referred to as HEPF (hydrophilically enhanced photothermal foam), is a three-dimensional (3D) framework of expanded graphite (EGr) enmeshed within a polymer network of polyurethane (PU) and poly(sodium acrylate) (PSA). We chose EGr as the solar absorber because of its abundance, low cost, intrinsic broad band light absorption, and high internal porosity, which allow the fabrication of a lightweight and self-floating foam with low thermal conductivity due to trapped air. The role of PU is critical for providing structural integrity to HEPF by acting as the amphiphilic “glue” that (i) binds the EGr granules together and (ii) bridges the hydrophilic PSA to the hydrophobic EGr,^43^ whereas the PSA network is essential for imparting moisture-capturing ability and enhanced water-transport properties to HEPF. Because of its enhanced hydrophilicity, the foams can be utilized not only for solar-driven desalination but also for the collection and utilization of water from the environment. For example, when water sources are available only intermittently, the foam can be used to absorb and store water, whereas in extreme circumstances where (liquid) water supply is totally absent, it can be used to harvest water via moisture capture followed by evaporation in a solar still. To the best of our knowledge, this is the first study that coherently incorporates all the desirable properties for solar evaporation, water collection, and atmospheric-water harvesting into a single integrated material.

## 2 Experimental Section

### 2.1 Fabrication of Photothermal Foams

HEPF was prepared by mixing 0.25 g of EGr (superexpanded graphite, Grafysorber, kindly offered by Directa Plus) with 4 g of waterborne aliphatic polyurethane (PU; Esacote PU77 from Lamberti S.p.A.; 35 wt % of PU and 65% of water)^44,45^ and a 5 mL aqueous solution containing 0.65 g of sodium acrylate (SA as monomer), 0.05 g of *N*,*N*′-methylenebis(acrylamide) (MBA, as cross-linker), 36.5 mg of ammonium persulfate (APS, as initiator), and 10 μL of *N*,*N*,*N*′,*N*′-tetramethylethylenediamine (TEMED, as accelerator). The admixture was then cast onto a Teflon dish and left to cure in the oven at 80 °C overnight. PU/EGr (or photothermal foam, PF) control samples were prepared in the same manner as HEPF, but without the addition of SA monomers. Briefly, 0.25 g of EGr with a 4 g dispersion of waterborne aliphatic PU (35 wt %) was mixed and cast onto a Teflon dish before it was left to cure in the oven at 80 °C overnight.

### 2.2 Materials Characterization

Morphology of foams coated with a layer of 10 nm Au was studied using a scanning electron microscope (SEM) (JEOL JSM-6490LA) at an acceleration voltage of 10 kV. The pore size distribution was characterized using Pascal 140 Evo and Pascal 240 Evo mercury intrusion porosimeters (Thermo Fisher Scientific). The chemical composition and interactions of components in the foams were studied using a single-reflection attenuated total reflection (ATR) accessory (MIRacle ATR, PIKE Technologies) coupled to a Fourier transform infrared (FTIR) spectrometer (Vertex 70v FT-IR, Bruker). All spectra shown were averaged from 128 repetitive scans recorded in the range from 3800 to 600 cm^–1^ with a resolution of 4 cm^–1^. Thermal images were recorded using an infrared camera (IR camera) FLIR A655sc, and subsequently analyzed using ResearchIR software. The optical absorption spectra (200–1800 nm) were obtained using a Cary JEOL UV–vis–NIR spectrophotometer. The thermal decomposition profiles and the first derivative curves were obtained through thermogravimetric analyses using a Q500 analyzer with a heating rate of 10 °C min^–1^, from 30 to 800 °C in a N_2_ atmosphere. The water contact angle was measured using a DataPhysics OCAH 200 contact angle goniometer. Thermal conductivity measurements were conducted using a TCi thermal conductivity analyzer.

### 2.3 Solar Evaporation and Desalination Experiments

Solar-evaporation tests were conducted at an ambient temperature of 19 ± 1 °C and a relative humidity (RH) of 40 ± 5%. Both PF and HEPF samples were prewetted in water for 0.5 h to ensure a steady-state water absorption rate and that they had swollen to their equilibrium diameters. The wet foams were then allowed to float on water in a Teflon beaker and illuminated under a solar simulator. The solar simulator used was a ScienceTech SLB-150B (Class BAA) with an AM1.5G air mass filter and was calibrated by Oriel reference solar cell and meter (91150 V). The mass loss of water was measured using an analytical balance (Kern, 0.01 mg accuracy). The final evaporation rates, *ṁ*, reported are the result of the subtraction of the evaporation rate of water in the dark from the measured evaporation rates under 1-Sun illumination. Solar-to-vapor conversion efficiency, η, was calculated using eq 1:^46−48^
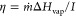
1where *I* is the power density of the incident light (1 kW m^–2^) and Δ*H*_vap_ is the evaporation enthalpy of water calculated as
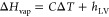
2where *C* is the specific heat capacity of water (4.2 kJ °C^–1^ kg^–1^), Δ*T* represents the temperature increase of water during vaporization, and *h*_LV_ is the latent heat of vaporization of water (2256 kJ kg^–1^ at 100 °C). Desalination experiments were conducted in a condensation chamber (Figure S1, Supporting Information) using synthetic seawater samples (3.5 wt %) prepared by dissolving sea salts (Sigma) in deionized water. The concentrations of Na, K, Ca, Mg, B, and Sr in the distillate were analyzed using an inductively coupled plasma optical emission spectrometer (ICP-OES) (iCAP 6300, ThermoScientific); samples were acidified with a few drops of concentrated HNO_3_ prior to analysis.

### 2.4 Water-Vapor Sorption–Desorption and Permeability Experiments

Dry HEPF samples were equilibrated in a climatic chamber set at 20 °C and the relative humidity (RH) was varied from 20 to 80%. The cyclic water-vapor sorption tests were conducted at 20 °C and 80% RH. To determine the water-vapor sorption under a saturated condition, we equilibrated a dry HEPF sample in a chamber saturated with water vapor (but not in contact with liquid water) for 72 h. The percentage of RH (% RH) of the chamber was monitored by Tinytag data loggers; 100% RH was achieved after 17 h of equilibration (Figure S2, Supporting Information). The percentage of absorbed water (from water vapor and liquid water) recovered via solar evaporation was calculated using the equation
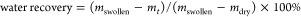
3where *m*_swollen_, *m*_dry_, and *m*_*t*_ are the masses of HEPF in the swollen state, in the dry state, and at time *t* during evaporation, respectively.

Water-vapor permeability (WVP) of the samples was determined at 20 °C and 100% RH gradient (ΔRH) according to the ASTM E96 standard method.^49,50^ A 100% RH gradient was created by placing a permeation chamber filled with 400 μL of deionized water (which generates 100% RH within the chamber) in a desiccator maintained at 0% RH using anhydrous silica gel. The foams were mounted on the permeation chambers and water-vapor transmission through the samples can be monitored from the mass loss of the permeation chamber as a function of time. The water-vapor permeability rate, WVP, can be calculated using the equation
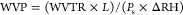
4where WVTR is the water-vapor transmission rate determined by taking the ratio of the rate of water evaporation from the chamber to the surface area of the foam exposed to water vapor, *L* is the thickness of the foam, and *P*_s_ is the saturation water-vapor pressure at 20 °C.

### 2.5 Characterization of Different Water States in Wetted Foams

The water states in the swollen foam samples were characterized according to a published method.^50,51^ Briefly, the polymer-bound water was quantified by the water-vapor adsorption process. In particular, a dry foam is placed in a chamber saturated with water vapor (but not in contact with liquid water) until its mass becomes constant. The mass increase due to water-vapor adsorption is the mass of the polymer-bound water. The amount of free water in the swollen foam (i.e., saturated with liquid water) was estimated by the mass decrease of the swollen foam after being subjected to vacuum suction on a microfilter unit at an absolute pressure of 100 kPa for 15 min. The mass of intermediate water was determined by measuring the difference between the mass of the deswollen foam after vacuum suction (i.e., summation of masses from dry foam + polymer-bound water + intermediate water) and the mass of the foam at the end of the water-vapor adsorption experiment (i.e., summation of the masses of dry foam + polymer-bound water). Note that both the polymer-bound water and intermediate water collectively constitute bound water (or nonassociated water) while free water can also be regarded as associated water.

## 3 Results and Discussion

### 3.1 Morphological and Chemical Properties of HEPF

**Figure 1:**
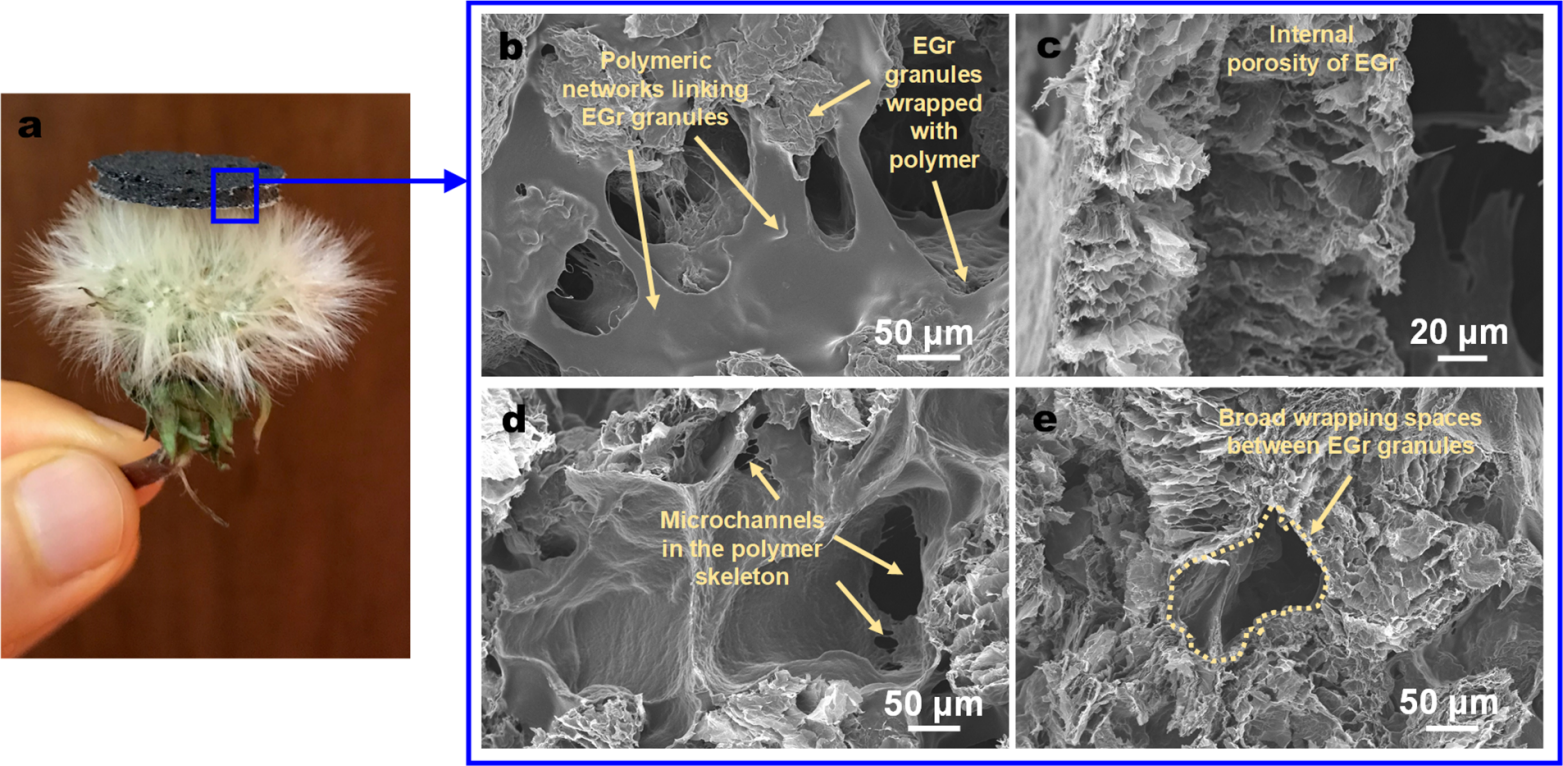
(a) Photograph showing a HEPF standing on a dandelion, demonstrating its lightweight property. SEM images showing the hierarchical pore structure from (b) the surface and (c–e) the cross section of the HEPF.

HEPF was prepared by polymerizing sodium acrylate in a mixture containing EGr granules and waterborne PU (which comprises aliphatic polycarbonate (PC) diol and aliphatic diisocyanate segments).^51^ As shown in Figure 1a, the as-synthesized HEPF appears black and is lightweight (bulk density of 0.2 g cm^–3^). A scanning electron microscopy (SEM) image of the surface shows an open porous network, where the individual EGr granules are connected and wrapped by a polymeric network with a smooth surface (Figure 1b). In particular, the external surface of the EGr is rough and wrinkled, while its internal surface is a multilayered structure of high porosity (Figure S3, Supporting Information). A morphological study of the cross section of HEPF reveals a hierarchical pore structure featuring the following: (i) submicrometer- to micrometer-sized internal pores of EGr granules, (ii) microchannels of diameters ranging from tens to hundreds of micrometers in the skeleton of the polymer matrix, and (iii) broad wrapping spaces (>100 μm) formed at the juncture between multiple polymer-enwrapped EGr granules (Figure 1c–e). Mercury intrusion porosimetry confirmed that HEPF has both micropores and macropores with bimodal pore sizes of 100 and 500 μm and a pore volume of 1.6 cm^3^ g^–1^ (Figure S4, Supporting Information). Note that, besides these physical pores, the polymeric networks also present nanoscale molecular meshes, whose sizes are influenced by the degree of cross-linking.^52−55^ Differential thermogravimetry and FTIR analyses indicate that the polymeric network consists of independent PU and PSA networks that interact mutually via extensive intermolecular hydrogen bonding (Figures S5 and S6, Supporting Information). In particular, hydrogen bonds are preferentially formed between the N–H of the urethane groups in PU with the carbonyls of the carboxylate groups of the PSA network, rather than with the carbonyl groups (mostly from urethane and some from polycarbonate groups) of another PU chain. As such, intimate contact between PU and PSA networks can be expected.

### 3.2 Photothermal Properties of HEPF

**Figure 2:**
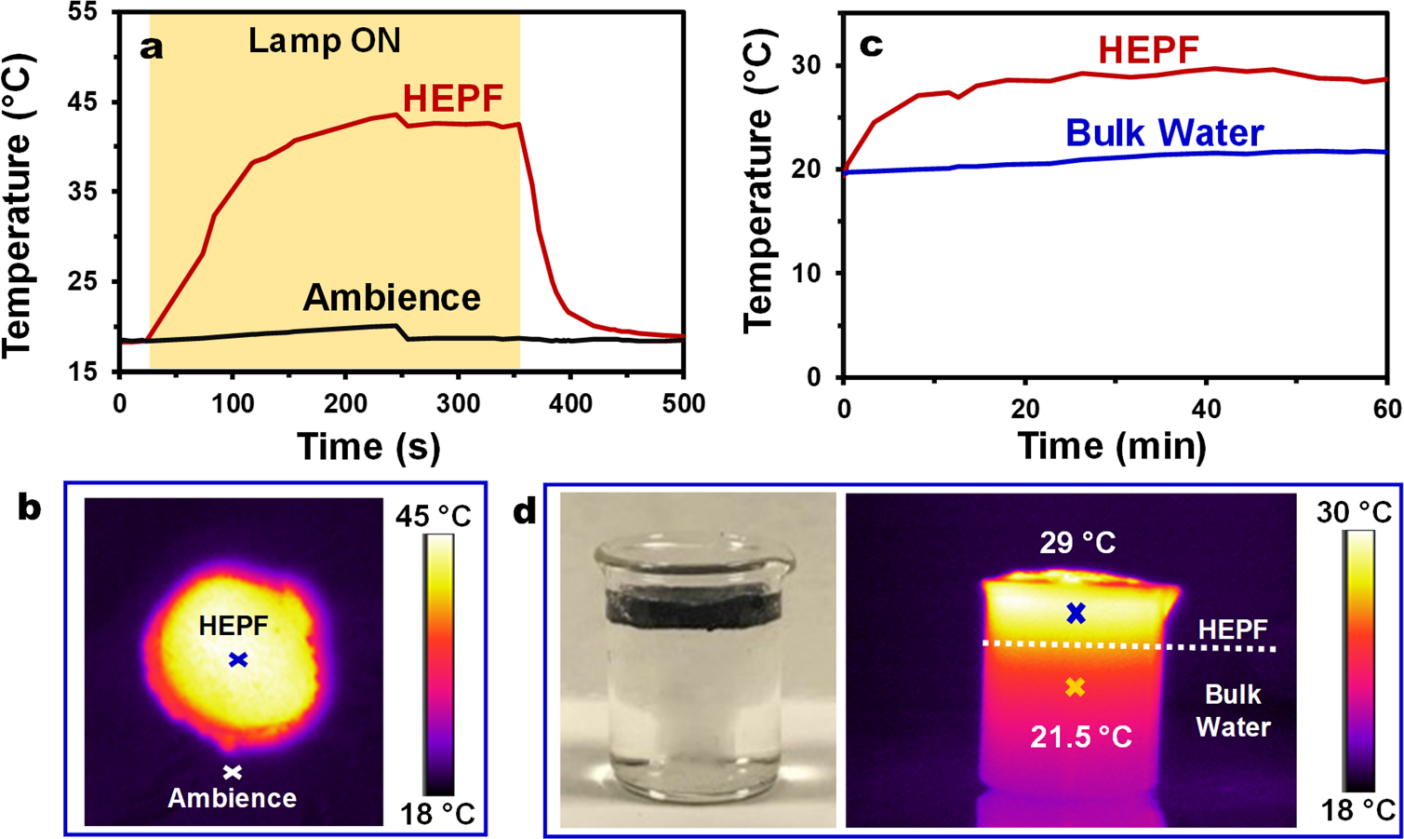
(a) Temperature profile of a dry HEPF under 1-Sun illumination and (b) the corresponding thermal image. (c) Temperature profile of HEPF versus bulk water and (d) the corresponding digital and thermal images.

The highly interconnected and porous network of HEPF combined with the intrinsic broad band light absorption property of EGr led to an exceptional light-harvesting ability. We found that a 2.5 mm thick HEPF shows complete light absorption with negligible transmittance and reflectance (Figure S7, Supporting Information). The high optical extinction of HEPF enables an effective solar–thermal energy conversion as evidenced by the rapid temperature increase by 25 °C within 2 min of 1-Sun illumination on a dry HEPF (Figure 2a,b). The thermal image and temperature profile of a prewetted HEPF floating on water suggest that the heat generated (after 1-Sun illumination on its surface) remains localized within the foam because the temperature of the bulk water (i.e., water underneath the foam) shows little change (Figure 2c,d). The heat localization effect may be attributed to the low thermal conductivity (0.039 ± 0.002 W m^–1^ h^–1^) of dry HEPF arising from the trapped air in the internal pores of EGr. Furthermore, the surface temperature of the (wet) HEPF remains relatively low (i.e., 29 °C versus 45 °C for the dry foam), indicating the efficiency of the evaporative cooling enabled by the effective heat transport in the presence of water (thermal conductivity of wet HEPF: 1.66 ± 0.08 W m^–1^ K^–1^). Note that the evaporative cooling effect also minimizes heat loss to the ambient environment (calculation details in section S2.5, Supporting Information).

### 3.3 Water Sorption–Desorption Properties of HEPF

**Figure 3:**
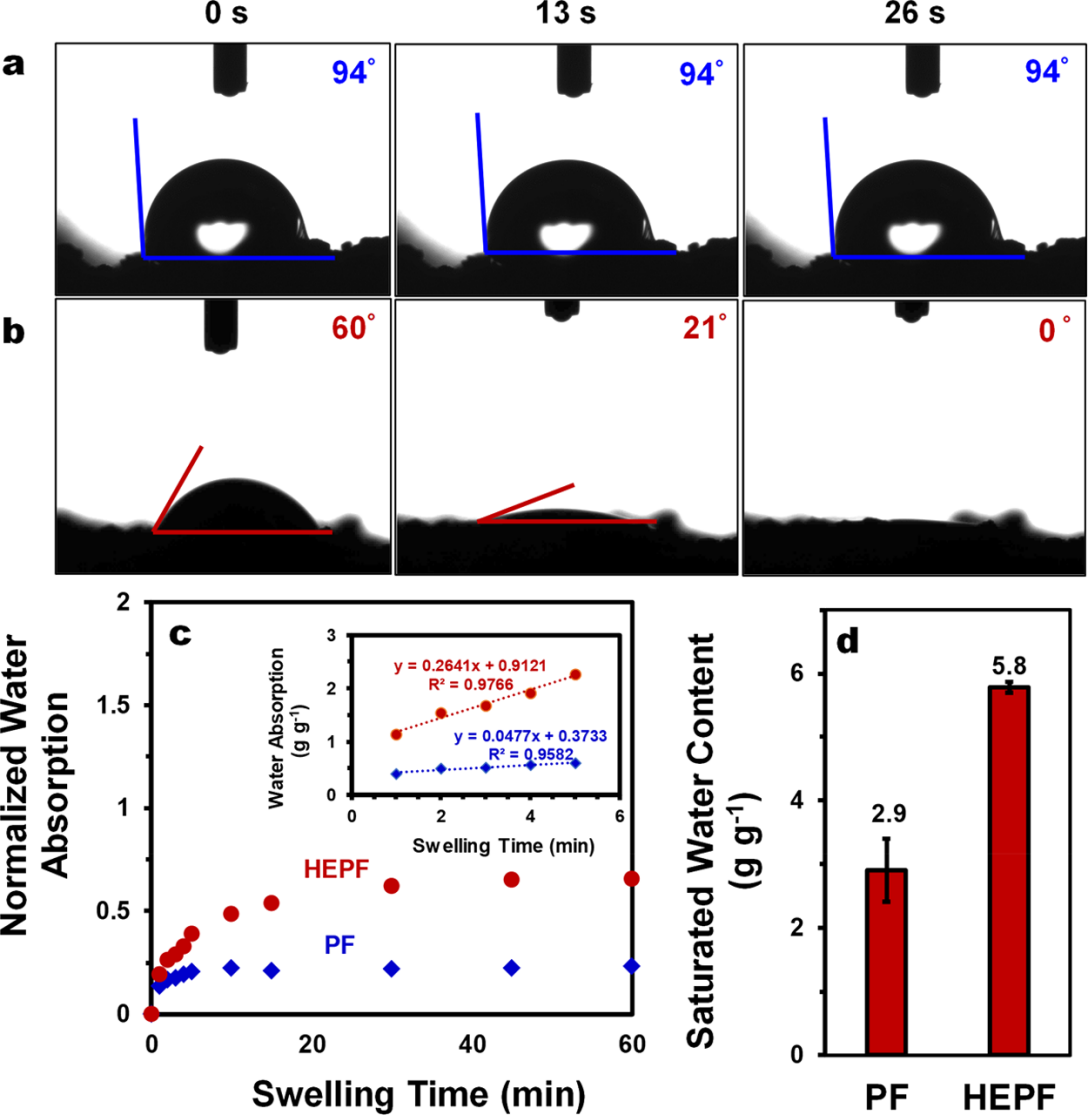
Static water contact angle measurements of (a) PF and (b) HEPF. (c) Time profiles of normalized water absorption with respect to saturated water content for PF and HEPF. (d) Saturated water contents in PF and HEPF (after 24 h equilibration in deionized water).

To investigate the effect of PSA hydrophilic enhancement in HEPF, we prepared a photothermal foam (PF) without PSA consisting of only PU and EGr. Compared to PF alone, the addition of a PSA network significantly improves the hydrophilicity of the resultant HEPF. As shown in Figure 3a, the water droplet remained for at least 26 s on the surface of the PF with an initial contact angle of 94°. In contrast, the more hydrophilic HEPF has an initial water contact angle of 60° and the droplet was gradually absorbed into the foam within 26 s (Figure 3b). Both PF and HEPF show a rapid increase in water uptake during the first 5 min of contact with water and reach a steady state within 30 min (Figure 3c). Significantly, the water-transport rate of HEPF is about 5.5 times higher than that of PF (0.264 vs 0.048 g of H_2_O/g of foam/min). In the equilibrium state, the water absorption capacity of HEPF is significantly higher compared to that of PF (5.8 vs 2.9 g of absorbed H_2_O/g of foam) (Figure 3c).

**Figure 4:**
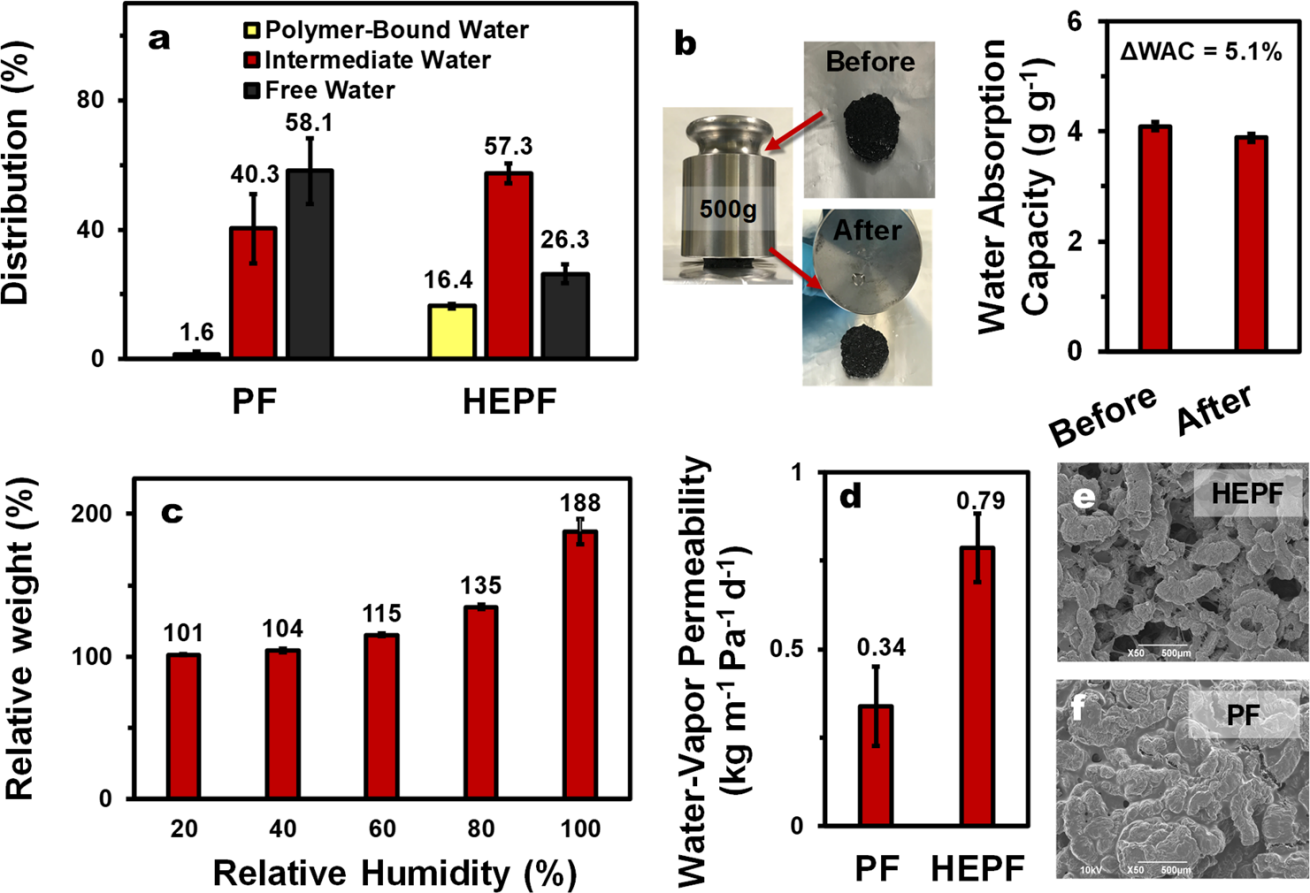
(a) Distribution of the different water states in PF and HEPF. (b) Effect of external loading (500 g) on the water absorption capacity of HEPF and the corresponding photographs. (c) Relative weight increase of HEPF after being placed in a climatic chamber at varying relative humidities. (d) Water-vapor permeability of HEPF vs PF. Topographic SEM images of (e) HEPF and (f) PF.

As shown in Figure 4a, the water present in HEPF and PF can exist in three different states, namely, (i) polymer-bound water, (ii) intermediate water, and (iii) free water. Both the polymer-bound water and intermediate water (or collectively referred to as bound water) are associated with the polymer via hydrogen bonds at varying degrees, and thus show completely different behaviors compared to bulk water. In contrast, free water is not associated with the polymer and thus its behavior is similar to that of bulk water.^56−58^ Both PF and HEPF contain a substantial amount of bound water, although the presence of a PSA network in HEPF results in a higher amount of polymer-bound water (16.4% vs 1.6%) and intermediate water (57.3% vs 40.3%) compared to that of PF. This strong interaction between PSA and water molecules enables HEPF to retain 95% of the amount of absorbed water even when placed under a 500 g load (Figure 4b) and to absorb water vapor up to 188% its initial mass (Figure 4c).

To ascertain whether the water vapor can efficiently pass through the foams, we measured the water-vapor permeability of both PF and HEPF, a parameter that indicates the ease to which water vapor passes through the horizontal plane of the foams when they are subjected to a humidity gradient. In fact, the water-vapor permeability is a significant parameter for the 3D solar–vapor generators, in particular, because the fact that boiling may take place in nonevaporative regions that are not directly exposed to air cannot be excluded. A higher water-vapor permeability would, thus, suggest that in the foams there are pathways from where the vapor bubbles migrate more easily from nonevaporative regions to the surface for evaporation, and is thus indicative of a high solar-evaporation efficiency. The results indicate that the water vapor can efficiently pass through the pores in HEPF and exit its surface when there is a humidity gradient, resulting thus in a high net evaporative effect (Figure 4d). Note that such a humidity gradient can be expected during solar evaporation as it has been recently shown that local spots of low humidity are generated on the surface of solar–vapor generators during the solar-evaporation process.^59^ In fact, the water-vapor permeability of the more-hydrophilic HEPF is almost twice that of the less-hydrophilic PF. This may be ascribed to the more open and interconnected network of HEPF with broad macropores, which are superior to the closed pores in PF as vapor escape channels (Figure 4e,f).

### 3.4 Application of HEPF for Solar-Driven Atmospheric-Water Harvesting and Desalination

**Figure 5:**
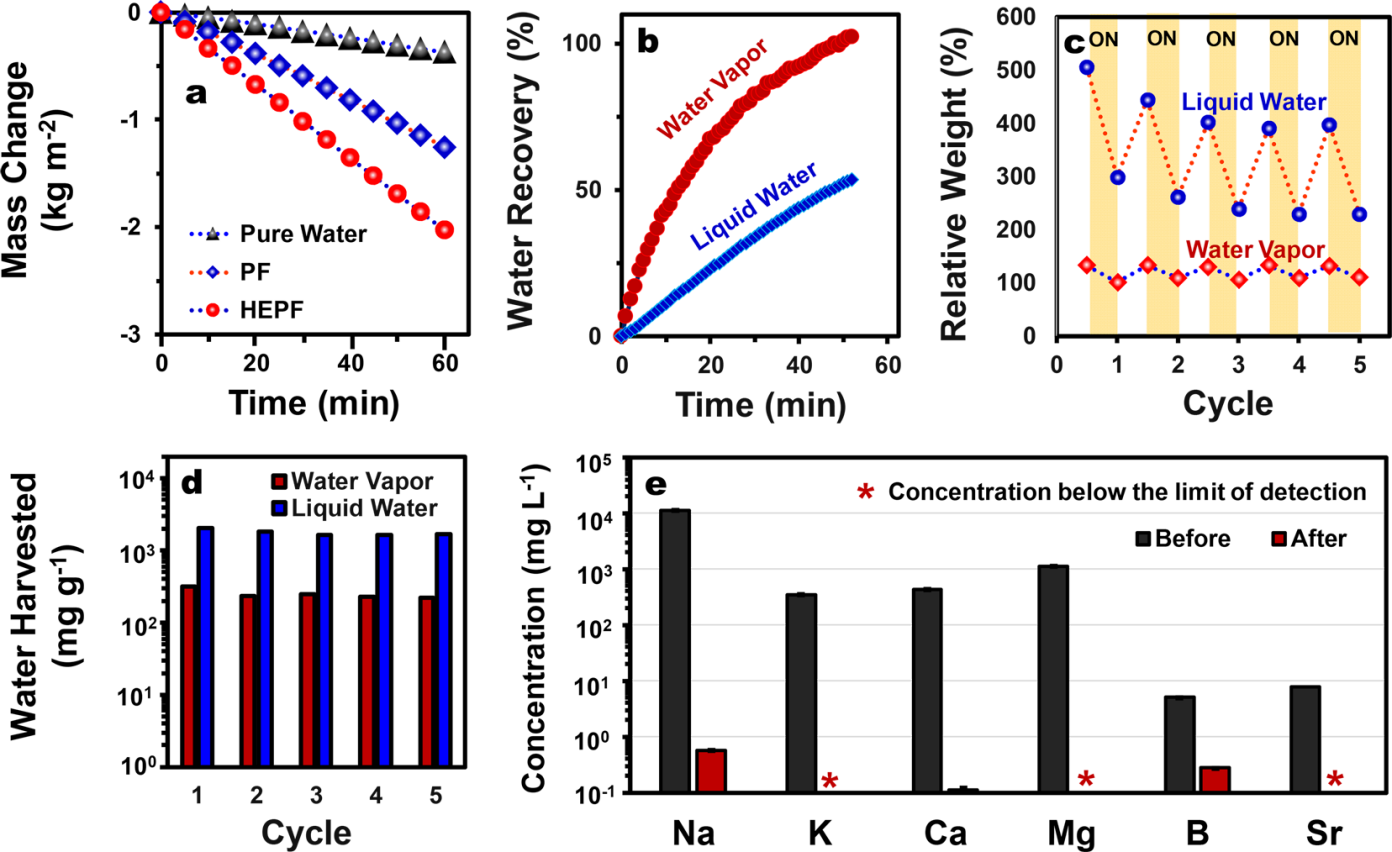
(a) Cumulative mass loss of water in the presence of and without the presence of the foams due to evaporation under 1-Sun illumination. (b) Percentage of the absorbed water recovered via solar evaporation under 1-Sun illumination of the HEPF. The water was absorbed in the HEPF either in the form of vapors (by equilibration in a climatic chamber at 80% relative humidity and 20 °C) or liquid water (by swelling in water for 5 min). (c) Relative weight variation of the HEPF upon hydration–dehydration cycles; for each cycle, in the hydration step (white region) the HEPF was either allowed to swell in water for 5 min (blue circles) or equilibrated in a climatic chamber at a relative humidity of 80% (20 °C) for 24 h (red diamonds), and in the dehydration step (yellow region) the HEPF was irradiated under the solar lamp (intensity of 1 Sun for 45 min). (d) The normalized amount of water harvested from HEPF (that has absorbed water in either liquid or vapor forms) for each hydration–dehydration cycle. (e) Concentration of the major elements in synthetic seawater (3.5 wt %) and in the distillate, as determined using inductively coupled plasma-optical emission spectrometry (ICP-OES).

As discussed in section 3.2, HEPF presents excellent photothermal properties, which suggests it can be applied for efficient potable-water production via a solar-driven process. The solar-evaporation rate, in particular, is a crucial parameter for evaluation of the feasibility of a material for such an application because it influences the quantity of purified water that can be produced after condensation. For determination of the solar-evaporation rate of the foams, PF and HEPF were individually floated on water and illuminated under 1 Sun after being prewetted in water for 0.5 h. The solar-evaporation rate (*ṁ*) of water through HEPF (after subtracting the evaporation rate of water in the dark field) is 1.89 ± 0.09 kg m^–2^ h^–1^, about 5.0 and 1.6 times higher than that of just water (0.38 kg m^–2^ h^–1^) and water through PF (1.17 ± 0.09 kg m^–2^ h^–1^), respectively (Figure 5a). Recently, the presence of polymer-bound water has been shown to improve the solar-evaporation efficiency by activating the intermediate water for evaporation via a lower-energy input as a result of a reduction in the equivalent vaporization enthalpy.^25,27,60^ As both HEPF and PF contain substantial amounts of bound water, we found that the equivalent vaporization enthalpy of water in both HEPF and PF is lowered by 20–27% compared to that of bulk water (Figure S8, Supporting Information). On the basis of the equivalent vaporization enthalpy of water in the foams (Δ*H*_vap_), the solar-to-vapor conversion efficiencies (η) of HEPF and PF, computed using eq 1, are 92.7 ± 4.4% and 68.7 ± 2.9%, respectively. The solar-evaporation results show that the performance of HEPF is among the best standalone solar evaporators reported to date (Figure S9 and Table S1, Supporting Information).

Considering the fact that the HEPF can absorb a substantial amount of water, both in the liquid and vapor forms, the possibility of using it as a moisture absorber to harvest water from the environment via a solar-driven humidification–dehumidification process is tested.^61−65^ The solar-driven humidification–dehumidification process involves the following: (i) exposure of the HEPF to humid air for water-vapor capture, followed by (ii) recovery of the captured moisture through solar evaporation in a condensation chamber. Figure 5b shows that the water vapor and liquid water absorbed by HEPF can be efficiently recovered under 1-Sun illumination. After 45 min of evaporation, about 97.2% and 48.1% of the absorbed water vapor and liquid water, respectively, were recovered through solar evaporation. This process can be repeated for over five cycles as HEPF can be repeatedly hydrated and dehydrated with minimal loss in the amount of water harvested (Figure 5c). Furthermore, the average amounts of absorbed vapor and liquid water that can be harvested were found to be 250 ± 39 and 1771 ± 187 mg of H_2_O/g of HEPF, respectively (Figure 5d).

The possibility of utilizing HEPF for solar-driven desalination was also investigated. To do so, the distillate collected from the condensation of vapor generated via solar evaporation of a synthetic seawater sample (3.5 wt %) was collected and the concentration of the major elements (Na, K, Ca, Mg, B, and Sr) was analyzed. As Figure 5e shows, the concentrations of various elements in the distillate are significantly reduced, rendering it suitable for potable use.^66^ In particular, the concentration of Na is reduced by at least 99.99%, while that of K, Mg, and Sr are below the instrumental detection limit.

To sum up, as demonstrated so far, HEPF is a versatile material, able to produce potable water not only from seawater but also from alternative sources such as atmospheric moisture. In fact, HEPF can be used to absorb and store water when water sources are available only intermittently, while in extreme circumstances where there is no liquid water supply they can be used to harvest water from the atmosphere via water-vapor capture followed by evaporation in a solar still. Such scenarios can be common in the aftermath of disasters whereby acute access to potable water may be critical for short-term sustenance and survival.

### 3.5 Plausible Mechanisms for Enhanced Solar Evaporation in HEPF

**Figure 6:**
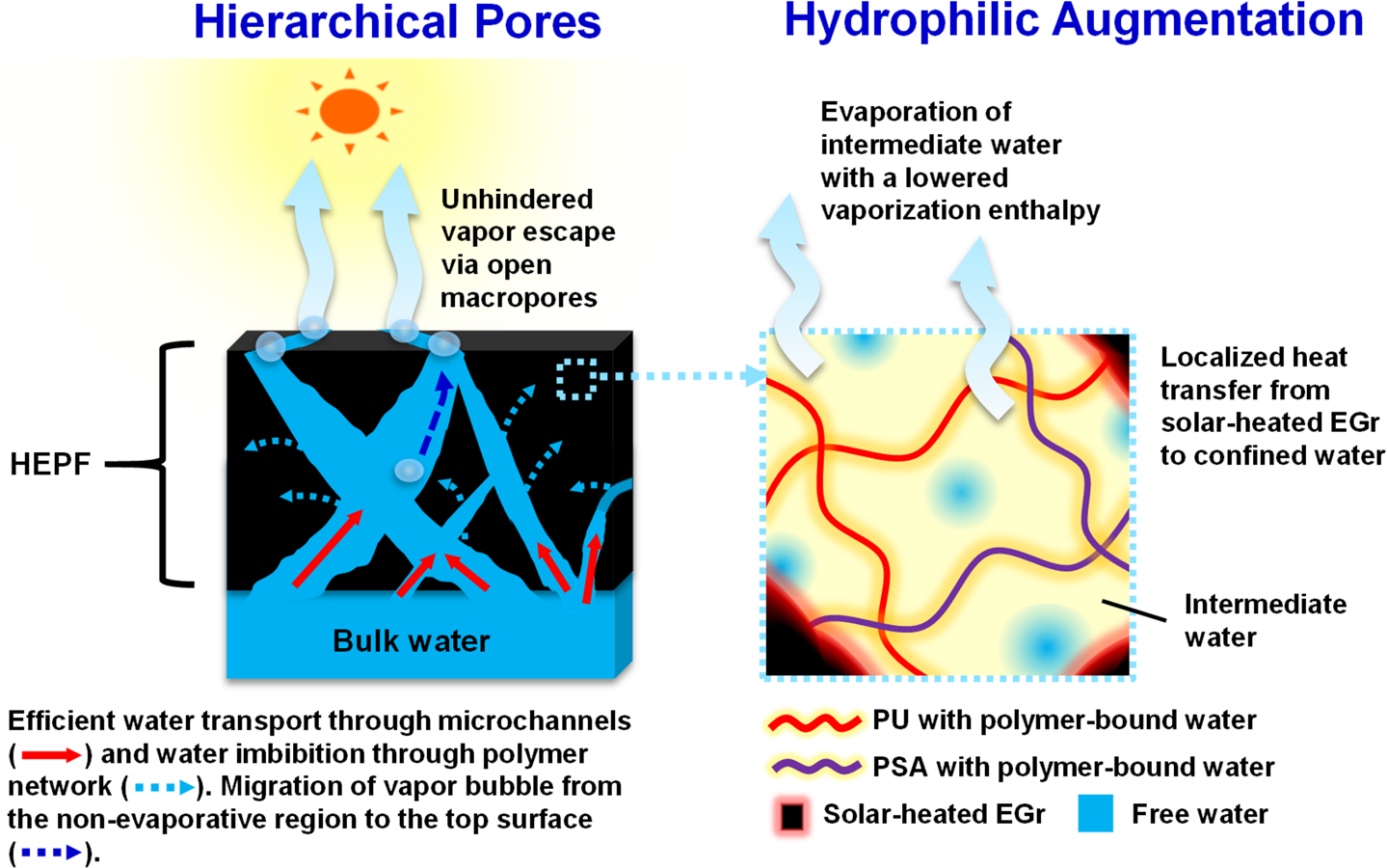
Mechanisms of enhanced solar-evaporation efficiency arising from the hierarchical pore structure (left panel) and hydrophilic augmentation (right panel) in HEPF.

The plausible mechanisms through which HEPF achieves high solar-evaporation efficiency involve its hierarchical pore structure and hydrophilic augmentation by PSA (Figure 6). The presence of PSA, in particular, results in a more open and interconnected network with broad pores in HEPF because of the electrostatic repulsion between the negative charges on PSA that leads to an expanded network.^58^ This facilitates efficient migration of vapor bubbles to the surface and its escape from the surface of HEPF, thereby preventing humidity buildup at the interface to enable efficient evaporation. Apart from this, hydrophilic augmentation by PSA in HEPF also results in improved water transport via (i) enhanced capillary effects through hydrophilic microchannels and (ii) water imbibition through polymer networks because of the high osmotic potential of PSA.^58,67,68^ The improved water-transport property of HEPF compared to that of PF is evidenced by its ability to absorb more water at a more rapid rate (Figure 3c,d). This rapid transport of a substantial amount of water can contribute to high vaporization efficiency by sustaining high heat-transfer rate through the following: (i) rapid water replenishment to “hot zones” for continuous evaporation and (ii) preventing the formation of insulating vapor films.^39,69−72^

Note that the “hot zones” in this case are localized hot regions that can exist between (i) the solar-heated EGr and the hydrated polymer networks and/or (ii) the solar-heated EGr and the (intermediate or free) water. As EGr is the solar absorber, it can be expected that the temperature on the solar-heated EGr is higher in all the above-mentioned cases, while it progressively decreases as the heat is being transferred to either the free water, the intermediate water, or the water bound to PU or PSA networks. As the hot zones are the areas at which heat transfer occurs, it is pertinent that there are good contacts between the solar-heated EGr and the water to be heated. Because of the hydrophobic nature of EGr, such intimate contact with the water may not be feasible. However, the presence of the amphiphilic PU increases the surface energy of the EGr, and therefore free water can readily wet it. For the same reason, good contact between the solar-heated EGr and the water bound to the PU network or the intermediate water in the vicinity of the PU network can be expected. The addition of PSA lowers the thermal contact resistance even further through the increase of the surface energy of the PU/EGr surface. This results in a more effective water uptake and the subsequent more efficient wetting of the PU and therefore of the solar-heated EGr. Thus, the heat-transfer rate in HEPF is expected to be higher compared to that of the PF. Indeed, the thermal conductivity of wetted HEPF was found to be significantly higher compared to PF (1.66 ± 0.08 vs 1.02 ± 0.05 W m^–1^ K^–1^).

The enhanced solar-evaporation efficiency of HEPF can also be attributed to the presence of polymer-bound water, which activates the intermediate water to evaporate at a lower-energy requirement by weakening the cohesive forces between the water molecules.^25,27,60,73^ As the presence of a PSA network in HEPF results in a higher amount of polymer-bound water and intermediate water compared to PF alone (Figure 4a), the higher solar-vaporization efficiency in HEPF may be ascribed to the presence of a higher amount of water existing in a state evaporable via a lower-energy input. Furthermore, the bound water, which is present in close proximity to the solar-heated EGr granules enwrapped within a highly vascularized polymeric network, exposes a small quantity of water to the hot zones on EGr. The intimate contact between the solar-heated EGr with the bound water of a smaller thermal mass in addition to the insulating effects from the polymer result in efficient concentration and transfer of heat, thereby inducing effective evaporation.

## 4 Conclusions

In conclusion, we report a rationally designed hydrophilic and self-floating foam that achieves a high solar-evaporation rate of 1.89 kg m^–2^ h^–1^ and a solar-to-vapor conversion efficiency of 92.7% under 1-Sun illumination. The excellent solar-evaporation efficiency of the foam may be attributed to the hierarchical pore structure and hydrophilic augmentation that improve both the heat- and water-transport efficiencies during the solar-evaporation process. In addition, the foam can be repeatedly hydrated and dehydrated through which 1 g of the foam can harvest 250–1770 mg of H_2_O via moisture absorption or water collection followed by solar evaporation in one hydration–dehydration cycle. These demonstrate the versatility of the foam in harvesting freshwater from various sources—not only seawater but also the atmospheric moisture, a previously untapped water resource. This can be a huge advantage in dealing with the suboptimal circumstances in acute emergencies where there is a high level of uncertainty regarding the availability and quality of source water. The lightweight of the foam, its versatility, and simple operation indicate that it can be a potent water solution for off-grid applications.

## Supporting Information Available

Method description for the fabrication of PSA/EGr foams; setups for the solar desalination experiment and the water-vapor saturated chamber; additional results (PDF)
