# Supplementary material for: Solar-Driven Freshwater Generation from Seawater and Atmospheric Moisture Enabled by a Hydrophilic Photothermal Foam
Source: ACS Appl Mater Interfaces. 2020 Feb 14;12(9):10307–16. doi: 10.1021/acsami.9b20291 (PMC7997105; doi:10.1021/acsami.9b20291)
Supplement: Supplementary file 1 — am9b20291_si_001.pdf [file am9b20291_si_001.pdf]

Electronic Supplementary Information

Solar-Driven Freshwater Generation from Seawater and Atmospheric Moisture Enabled by a Hydrophilic Photothermal Foam

Siew-Leng Loo,‡*[a], Lía Vásquez Sánchez,‡[ab] Uttam C. Paul,[a] Laura Campagnolo,[ab] Athanassia Athanassiou,[a] and Despina Fragouli*[a]

[a]*Smart Materials, Istituto Italiano di Tecnologia, Via Morego 30, 16163 Genoa, Italy*
[b]*Dipartimento di Chimica e Chimica Industriale (DCCI) Università degli Studi di Genova, Via Dodecaneso 31, 16146 Genoa, Italy*

*Correspondence to: Despina.Fragouli@iit.it; Siew.Loo@iit.it
†Authors with equal contribution.

S1. Supplementary Methods

S1.1 Fabrication of Photothermal Foams
S1.2 Setup for Solar Desalination Experiments
S1.3 Equilibration of the Water-Vapor Saturated Chamber

S2. Supplementary Figures

S2.1 Morphology of EGr
S2.2 Pore Size Distribution of HEPF
S2.3 Chemical Properties and Interaction Between PU and PSA in HEPF
S2.4 Photothermal Properties of HEPF
S2.5 Heat Loss Analysis
S2.6 Estimation of Equivalent Enthalpies of Vaporization of Water
S2.7 Performance Comparison with Other Solar Evaporators
S2.8 Water Retention Property of HEPF

S1 Supplementary Methods

S1.1 Fabrication of PSA/EG (control)

PSA/EGr control samples were prepared in the same manner as HEPF, but without the addition of PU. Briefly, 0.25 g of EGr was mixed with a 5-mL aqueous solution containing 0.65 g of SA, 0.05 g of MBA, 36.5 mg of APS, and 10 µL of TEMED that was then casted on Teflon dish and left to cure in the oven at 80 °C overnight. Note that such control samples broke apart upon swelling in water due to the strong unbounded swelling forces of PSA networks. Further characterization involving contact of PSA/EGr samples with water was not conducted because of this reason.

S1.2 Setup for Solar Desalination Experiments

Desalination experiments were conducted in a condensation chamber as shown in Figure S1.

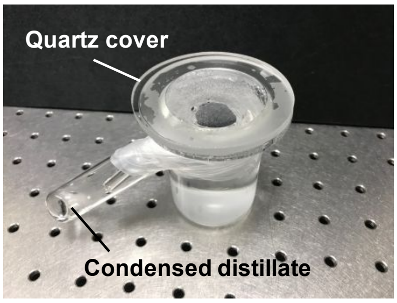

Figure S1 The condensation chamber used for collection of distillate.

S1.3 Equilibration of the Water-Vapor Saturated Chamber

Figure S2 shows the temperature and relative-humidity profile of the water-vapor saturated chamber used for water-vapor sorption experiments. The results show that 100% RH was achieved after 17 h of equilibration.

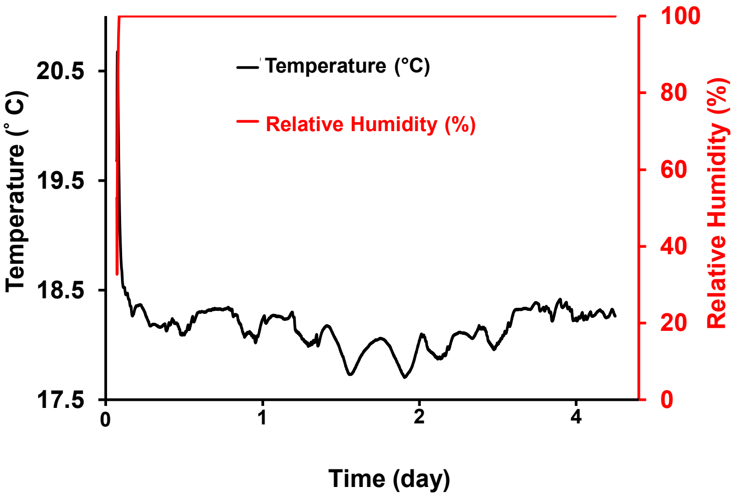

Figure S2 Temperature and relative humidity profiles of a water-vapor saturated chamber.

S2 Supplementary Figures

S2.1 Morphology of EGr

Figure S3a shows that the EGr granules used to prepare HEPF have an average length of 1.99±0.82 mm and diameter of 0.24±0.06 mm (measured using ImageJ). Under a higher magnification, the SEM image revealed that EGr granules have a rough and wrinkled surface (Figure S3b), while the SEM image of the cross-section shows the multi-layered structure of the internal pores (Figure S3c).

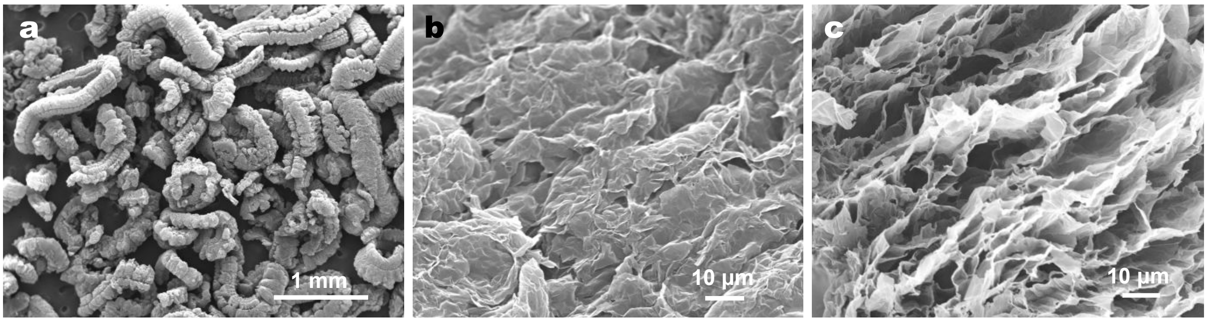

Figure S3 SEM images of EGr granules showing (a) the overall surface morphology under low magnification, (b) surface roughness under high magnification, and (c) the internal pores present in a cross-sectioned granule.

S2.2 Pore Size Distribution of HEPF

The pore size distribution of HEPF shows a bimodal distribution with modal peaks at 100 and 500 microns (Figure S4).

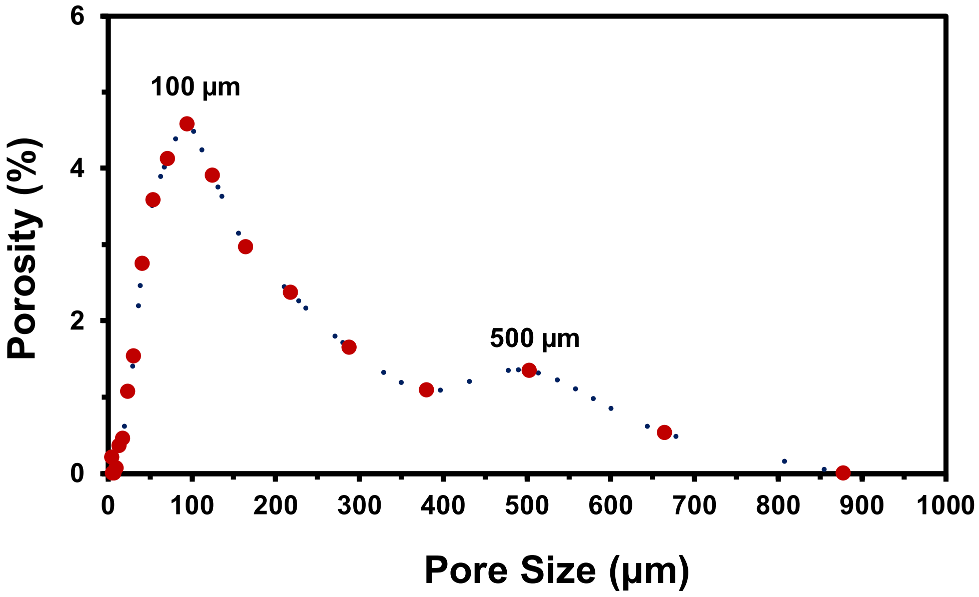

Figure S4 Pore size distribution of HEPF as determined from mercury intrusion porosimetry.

S2.3 Chemical Properties and Interaction Between PU and PSA in HEPF

Figure S5 shows that the major decomposition temperatures of PU/EGr and PSA/EGr are at 200-400 °C and 400-500 °C, respectively. The two characteristic decomposition temperatures are still present in the differential thermogravimetry (DTG) pattern of HEPF indicating that both PU and PSA are present as individual networks within the 3D scaffold. Nonetheless, we note that there is a slight shift in the decomposition peaks (attributed to PU) in the DTG pattern of HEPF whereby the second decomposition peak shifts from 330 °C to 290 °C with a concurrent increase in the decomposition rate of the first decomposition peak at 210 °C. The PU used in this study was made from aliphatic prepolymers of polycarbonate (PC) diol and aliphatic diisocyanate. As such, the first degradation step at 210 °C is attributed to the hard segment in PU that is primarily comprising urethane bonds, while the second decomposition step is due to degradation of the soft segment mainly consisting of the PC diol. [1-2] The increase of the decomposition rate of urethane bonds may be due to the presence of anionic carboxylate groups (from PSA) that prevents extensive urethane-urethane hydrogen bonding.[3] This reduces the phase separation between hard and soft segments preventing the shielding of the oxidizable carbonyl groups in the PC backbone thereby resulting in a lower degradation temperature of the soft segment. The effect of PSA on the hydrogen bonding between the hard and soft segments in PU described here is consistent with the findings from FTIR analysis that will be described hereafter.

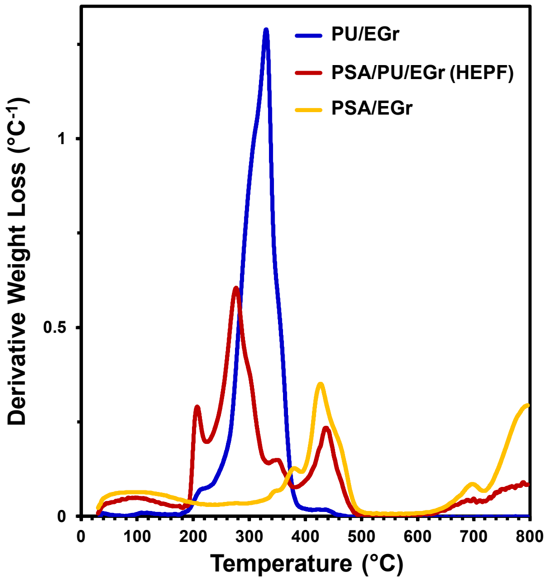

Figure S5 Derivative thermogravimetry (DTG) patterns for HEPF and control samples of PU/EG and PSA/EG.

FTIR analysis was conducted to investigate the chemical interaction(s) between PU and PSA. Note that because EGr shows strong IR absorption that interferes with the characteristic peaks of the organic polymers, FTIR samples were prepared without the addition of EGr. As such, an FTIR sample representing the interaction between PU and PSA in HEPF was prepared by polymerizing a solution of sodium acrylate (with crosslinker, initiator and accelerator) in the presence of waterborne PU via curing at 80°C; the sample is referred to as PSA/PU hereafter. Controls consisting of only PSA and PU were used for comparison reasons. The FTIR absorption spectra of PU, PSA and PSA/PU are shown in Figure S6.

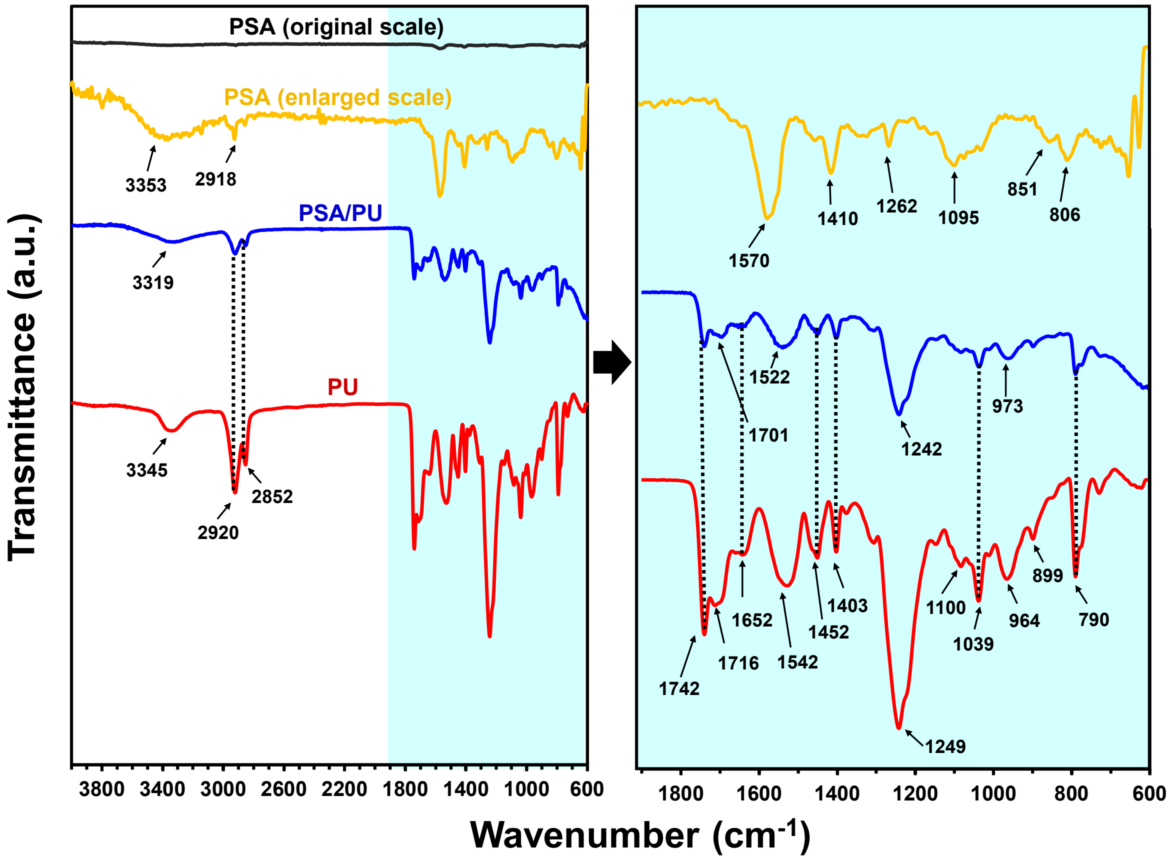

Figure S6 FTIR spectra of PSA, PU and PSA/PU.

For PSA samples, two strong absorption peaks were observed at around 1570 and 1410 cm^{-1}, which are assigned to the C=O stretching vibration of the carboxylate group (–COO^{-}). The peaks at 1262 and 1095 cm^{-1} correspond to the C–O stretching vibration. The absorption bands at 2918 cm^{-1} can be assigned to –CH$_2$– stretching vibration while the broad band at 3353 cm^{-1} may be attributed to O–H stretching vibration of water hydrogen-bonded to PSA.[4] For PU, the presence of carbonate (as PC diol in the soft segment of PU) is evidenced by the absorption bands of the group at 1453-1400 cm^{-1} and 899 cm^{-1}.[5] Accordingly, the C=O stretching vibration in the amide-I region (1630-1730 cm^{-1}) can be resolved into four distinct spectral features due to the degree of carbonyl H-bonding that can be distinguished as: (i) free (i.e, non-hydrogen-bonded) carbonyl groups in carbonates (1742 cm^{-1}), (ii) associated (i.e., hydrogen-bonded) carbonyl groups in carbonates (1716 cm^{-1}), (iii) free carbonyl groups in urethane (1700) and (iv) associated urethane (1652 cm^{-1}). [6-7] The strong absorption peaks at 1249, 1039, 790 cm^{-1} can be attributed to asymmetric bending of –COO, C–O stretching and C–O–C asymmetrical stretching, respectively. The absorption band at 3345 cm^{-1} signifies the presence of an aliphatic secondary amine N–H stretching combined with hydrogen bonding (in the urethane unit), and the corresponding C–N stretching and –NH in-plane bending are indicated by the absorption band at 1522 cm^{-1}. Furthermore, the two peaks at 2920 and 2852 cm^{-1} are due to methylene C–H asymmetric and symmetric stretching (in the PC backbone), respectively. The FTIR spectrum of PSA/PU resembles that of PU because the signal from PSA is very low compared to PU. As such, the spectrum of PSA/PU was compared to that of PU in order to identify changes to the chemical environment due to the presence of PSA. The

first difference in their spectra is the broadening and lowering of N–H stretching band frequency to 3319 cm^{-1} indicating their extensive involvement in hydrogen bonding. Furthermore, the absorption band at 1716 cm^{-1} (attributed to associated carbonates) disappeared in conjunction with the appearance of a lower-frequency peak at 1701 cm^{-1} attributable to free urethane groups. The asymmetric bending of –COO has also shifted to a lower frequency (1249 to 1242 cm^{-1}). Also, C–N stretching and –NH in-plane bending band frequency has shifted to a lower frequency (1542 to 1522 cm^{-1}). Taken together, we interpret these observations as a transformation of the hydrogen bonds between the N–H groups of urethane with the carbonyl groups (mostly from urethane and some from carbonate) between PU chains to hydrogen bonds being preferentially formed between the N–H groups of urethane in PU with the carbonyl groups of carboxylate (that is present in 1:1 molar ratio with every SA repeat unit) in PSA. This explains the disappearance of associated carbonyl in carbonates and the increased free carbonyl in urethane, which also corroborates with the findings from the DTG analysis. In view of the extensive hydrogen bonding between PU and PSA, intimate connections between the two networks can be expected.

S2.4 Photothermal Properties of HEPF

The transmittance and reflectance of a 2.5 mm thick HEPF were found to be negligible (Figure S7). The complete light absorption of HEPF may be attributed to the multiple internal reflectance that increased the optical path length and residence time within the 3D framework thereby increasing the probability of light absorption.

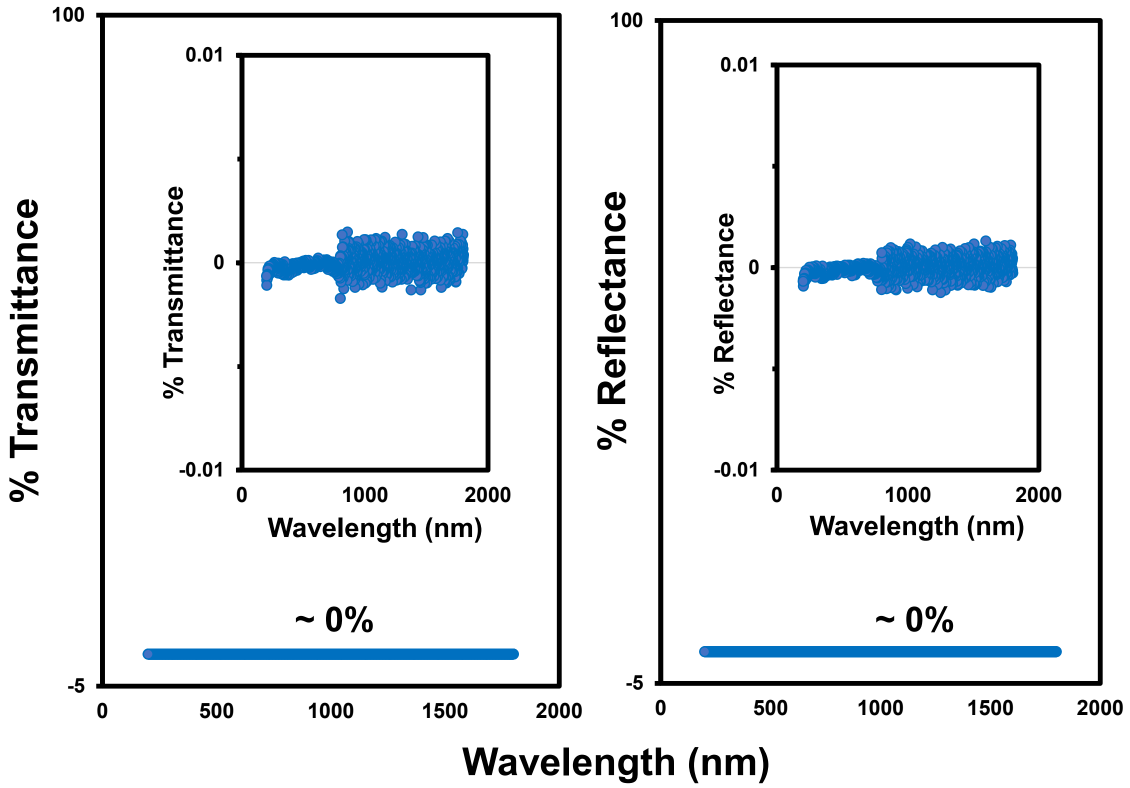

Figure S7 Optical transmittance and reflectance spectra of HEPF. Note that the insets show enlarged scale of the respective figures.

S2.5 Heat Loss Analysis

The possible heat dissipation processes in a solar evaporator may occur via (i) conduction, (ii) radiation and (iii) convection. The power input (I_{in}) in our evaporation experiments was 1 kW m^{-2}.

Conductive heat loss

Conductive heat flux (J_{cond}) from HEPF to the bulk water can be determined based on the following equation:

$$J_{cond} = Cm\Delta T_{bulk} = 39.9 \text{ J} \qquad \text{Equation (S1)}$$

Where C is the specific heat capacity of water (4.2 kJ °C^{-1} g^{-1}), m represents the weight of bulk water (5 g), and ΔT_{bulk} (21.5 – 19.6°C) represents the increased temperature of the bulk water after stable steam generation.

Energy input during solar evaporation, $E_{in} = I_{in} \times A \times t = 936$ J

Where A is the irradiated area of the foam (0.00026 m^2) and t is the time duration of the solar evaporation (3600 s).

Conductive heat loss = $J_{cond}/E_{in} \times 100\%$ = 39.9 J/936 J × 100% = **4.3%**

Radiative heat loss

Radiative heat transfer can be calculated using the Stefan-Boltzmann equation:

$$\Phi = \varepsilon A\sigma\,(T_{HEPF}^4 - T_{ambient}^4) \qquad \text{Equation (S2)}$$

Whereby Φ denotes the heat flux, ε is the emissivity, A is the area of the evaporation surface (0.00026 m^2), σ is the Stefan-Boltzmann constant (5.67×10^{-8} W m^{-2} K^{-4}), T_{HEPF} and $T_{ambient}$ are the temperatures of HEPF (302 K) and ambience (292 K), respectively. Since HEPF has an absorption, $\alpha = 1$ (transmission, $\tau = 0$ and reflection, $\rho = 0$), it can be treated as a black-body in thermal equilibrium with an ε of 1.

Radiation flux, $J_{rad} = \Phi/A = \varepsilon\sigma\,(T_{HEPF}^4 - T_{ambient}^4) = 59.4$ W m^{-2}

Radiative heat loss = $J_{rad}/I_{in} \times 100\%$ = 59.4 W m^{-2}/1000 W m^{-2} × 100% = **5.9%**

Convective heat loss

Convective heat transfer, P_{conv} can be calculated by the Newton's law of cooling:

$$P_{conv} = hA(T_{HEPF} - T_{ambient}) \qquad \text{Equation (S3)}$$

Where h is the convective heat transfer coefficient (5 W m^{-2} K^{-1}).[3, 18]

Convective flux, $J_{conv} = P_{conv}/A = h(T_{HEPF} - T_{ambient}) = 50$ W m^{-2}

Convective heat loss = $J_{conv}/I_{in} \times 100\%$ = 50 W m^{-2} /1000 W m^{-2} × 100% = **5%**

Therefore, the total heat loss of HEPF is **15.2%** (4.3% + 5.9% + 5%) under 1-Sun illumination.

S2.6 Estimation of Equivalent Enthalpies of Vaporization of Water

The total vaporization enthalpies of the water present in PF and HEPF were determined by comparing the intrinsic evaporation rates of pure water ($\dot{m}_{bulk\ water}$) with the intrinsic evaporation rates of the water in the absorbers ($\dot{m}_{absorber}$).[8] The determination of the intrinsic evaporation rates were conducted by simultaneously placing all samples in a closed chamber (at 20 °C and 40 % RH) in the dark and measuring their respective mass losses due to water evaporation. The equivalent total evaporation enthalpy of the water present in the absorber ($\Delta H_{vap,absorber}$) was estimated by assuming identical power input, (U_{in}) required for water vaporization[8]:

$$U_{in} = \Delta H_{vap,bulk\ water}\dot{m}_{bulk\ water} = \Delta H_{vap,absorber}\dot{m}_{absorber} \qquad \text{Equation (S4)}$$

Where $\Delta H_{vap,bulk\ water}$ is the evaporation enthalpy of bulk water (see Equation 2 in the main paper), and $\dot{m}_{bulk\ water}$ and $\dot{m}_{absorber}$ are the evaporation rates of bulk water and of the water present in the absorbers (PF or HEPF), respectively. The results indicated that the enthalpy of vaporization of PF and HEPF are 20-27% lower compared to that of bulk water (Figure S10). As proposed by Zhao et al., the presence of bound water in polymeric networks could lead to a reduction in the latent heat of evaporation due to the formation of water clusters that tend to evaporate more easily.[8] As shown in Fig. S9, both PF and HEPF contain a significant proportion of bound water existing as water molecules hydrogen bonded to the polymer within the 3D matrix and as intermediate water weakly interacting with the polymer (possibly at the pore wall interface). The strong hydrogen bonds between the (bound) water molecules with the carboxylate and urethane/carbonate groups in PSA and PU respectively, may have reduced the cohesive forces between water molecules. This activates the water molecules to escape via a lower-energy evaporation route through the formation of clusters.[8-10]

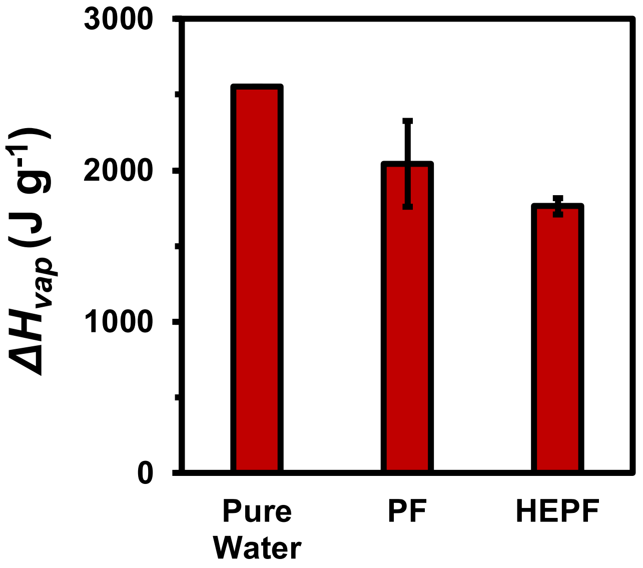

Figure S8 Equivalent vaporization enthalpies of waters in bulk water, PF and HEPF.

S2.7 Performance Comparison with Other Solar Evaporators

As shown in Figure S9, HEPF is ranked among the best stand-alone evaporators that show high evaporation efficiency without the requirement of additional external insulation or water-wicking mechanisms. A more detailed list for performance comparison that includes evaporators using external mechanisms is as shown in Table S1.

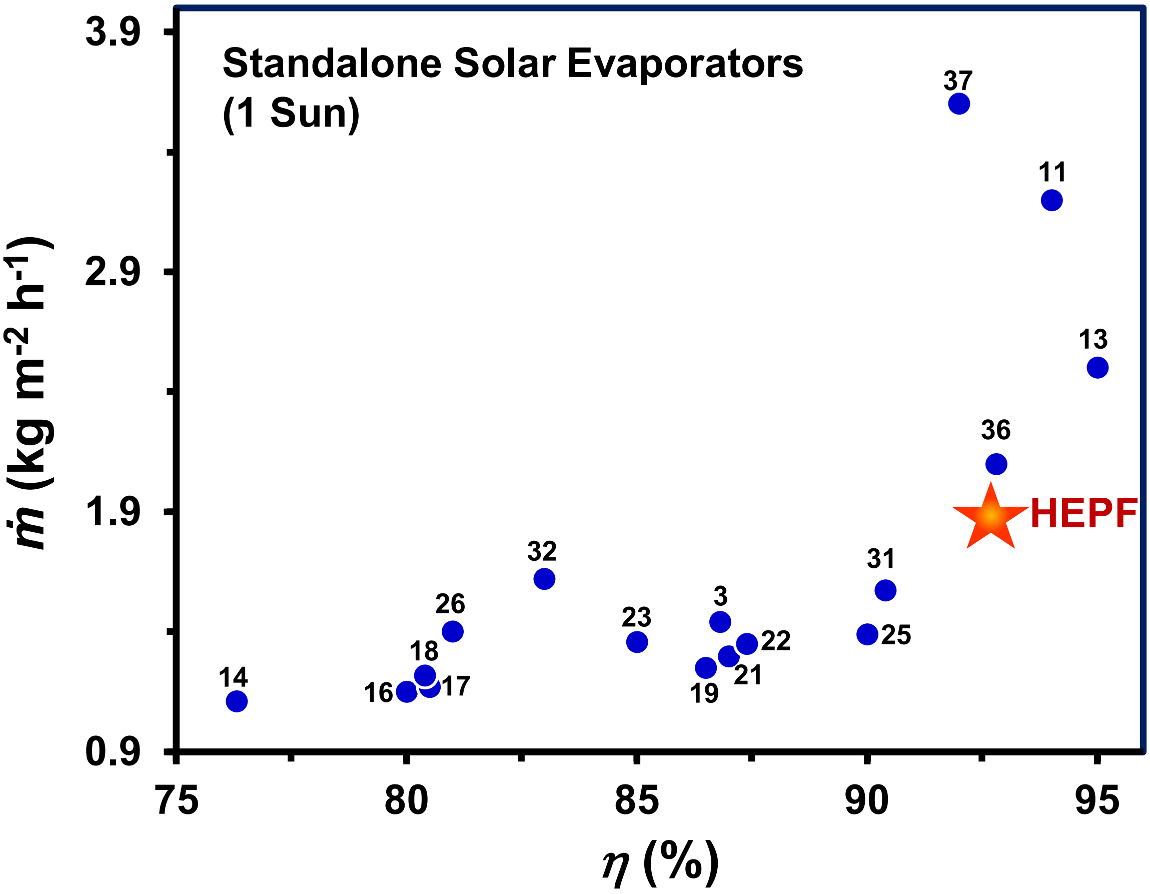

Figure S9 Performance comparison of the evaporation rate between HEPF with other standalone solar evaporators; note that the numbers in the figure are the references from which the data were extracted.

Table S1 Performance summary of recent high-efficiency solar evaporators

Solar Absorber Materials	$\dot{m}$ (kg m^{-2}h^{-1})	η (%)	Reference
CNF-CNT aerogel[a]	1.11	76.3	11
Mesoporous wood with coated graphite layer[a]	1.15	80	12
Polymerized oligoaniline foam[a]	1.17	80.5	13
rGO-MWCNT[a]	1.22	80.4	14
All in one evaporator[a]	1.25	86.5	15
CB/PMMA/PAN janus absorber	1.3	72	16
3D graphene foam[a]	1.3	87	17
Selenium-coated tellurium nanomaterials	1.32	86	18
CNT/CNC-coated PDMS foam[a]	1.35	87.4	19
Silica/Au composite gel[a]	1.36	85	20
Multilayer PPy nanosheets	1.38	92	21
N-enriched C sponge[a]	1.39	90	22
CMP aerogel[a]	1.4	81	23
pPEGDA-PANI double network gel	1.4	91.5	24
Hollow-CNT aerogels[a]	1.44	86.8	25
GO paper	1.45	80	26
Carbonized mushroom	1.475	78	27
MOF	1.5	96	28
Bilayer polypyrrole/melamine foam[a]	1.574	90.4	29
Vertically aligned graphene sheets	1.62	86.5	30
GO Aerogel[a]	1.622	83	31
Cu_2SnSe_3 or $Cu_2ZnSnSe_3$ infiltrated cellulose membrane	1.657	86.6	32
**HEPF**[a]	**1.89**	**92.7**	**This study**
MoS_2/C/polyurethane sponge	1.95	88	33
CNT-coated PAAM aerogel	2.00	85.7	34
Porous graphene skeleton frame	2.01	91.7	35
Ag-PSS-agarose/agarose[a,b]	2.10	92.8	36
PVA/CNT[a]	2.5	95	10
PVA/PPy[a]	3.2	94	8
PVA/Chitosan/PPy[a]	3.6	92	37

Note:
[a] Standalone systems
[b] Non-floatation mode

References

(1) Cakić, S. M.; Špírková, M.; Ristić, I. S.; B-Simendić, J. K.; M-Cincović, M.; Poręba, R. The waterborne polyurethane dispersions based on polycarbonate diol: Effect of ionic content. *Materials Chemistry and Physics* **2013,** *138* (1), 277-285.

(2) Cakic, S.; Lacnjevac, C.; Rajkovic, M. B.; Raskovic, L.; Stamenkovic, J. Reticulation of Aqueous Polyurethane Systems Controlled by DSC Method. *Sensors (Basel)* **2006,** *6* (5), 536-545.

(3) Król, P. Synthesis methods, chemical structures and phase structures of linear polyurethanes. Properties and applications of linear polyurethanes in polyurethane elastomers, copolymers and ionomers. *Progress in Materials Science* **2007,** *52* (6), 915-1015.

(4) Liu, C.; Yu, L.; Zhang, Y.; Zhang, B.; Liu, J.; Zhang, H. Preparation of poly(sodium acrylate-acrylamide) superabsorbent nanocomposites incorporating graphene oxide and halloysite nanotubes. *RSC Advances* **2013,** *3* (33), 13756-13763.

(5) Coates, J. J. Interpretation of Infrared Spectra, A Practical Approach in Encyclopedia of Analytical Chemistry R.A. Meyers (Ed.) **2000**, pp. 10815–10837

(6) Wang, F. C.; Feve, M.; Lam, T. M.; Pascault, J.-P. FTIR analysis of hydrogen bonding in amorphous linear aromatic polyurethanes. I. Influence of temperature. *Journal of Polymer Science Part B: Polymer Physics* **1994,** *32* (8), 1305-1313.

(7) Gomez, C. M.; Gutierrez, D.; Asensio, M.; Costa, V.; Nohales, A. Transparent thermoplastic polyurethanes based on aliphatic diisocyanates and polycarbonate diol. *Journal of Elastomers & Plastics* **2016,** *49* (1), 77-95.

(8) Zhao, F.; Zhou, X.; Shi, Y.; Qian, X.; Alexander, M.; Zhao, X.; Mendez, S.; Yang, R.; Qu, L.; Yu, G. Highly efficient solar vapour generation via hierarchically nanostructured gels. *Nature Nanotechnology* **2018,** *13* (6), 489-495.

(9) Kudo, K.; Ishida, J.; Syuu, G.; Sekine, Y.; Ikeda-Fukazawa, T. Structural changes of water in poly(vinyl alcohol) hydrogel during dehydration. *The Journal of Chemical Physics* **2014,** *140* (4), 044909.

(10) Zhou, X.; Zhao, F.; Guo, Y.; Zhang, Y.; Yu, G. A hydrogel-based antifouling solar evaporator for highly efficient water desalination. *Energy & Environmental Science* **2018,** *11* (8), 1985-1992.

(11) Jiang, F.; Liu, H.; Li, Y.; Kuang, Y.; Xu, X.; Chen, C.; Huang, H.; Jia, C.; Zhao, X.; Hitz, E.; Zhou, Y.; Yang, R.; Cui, L.; Hu, L. Lightweight, Mesoporous, and Highly Absorptive All-Nanofiber Aerogel for Efficient Solar Steam Generation. *ACS Applied Materials & Interfaces* **2018,** *10* (1), 1104-1112.

(12) Li, T.; Liu, H.; Zhao, X.; Chen, G.; Dai, J.; Pastel, G.; Jia, C.; Chen, C.; Hitz, E.; Siddhartha, D.; Yang, R.; Hu, L. Scalable and Highly Efficient Mesoporous Wood-Based Solar Steam Generation Device: Localized Heat, Rapid Water Transport. *Advanced Functional Materials* **2018,** *28* (16), 1707134.

(13) Chen, Q.; Pei, Z.; Xu, Y.; Li, Z.; Yang, Y.; Wei, Y.; Ji, Y. A durable monolithic polymer foam for efficient solar steam generation. *Chemical Science* **2018,** *9* (3), 623-628.

(14) Wang, Y.; Wang, C.; Song, X.; Megarajan, S. K.; Jiang, H. A facile nanocomposite strategy to fabricate a rGO–MWCNT photothermal layer for efficient water evaporation. *Journal of Materials Chemistry A* **2018,** *6* (3), 963-97.

(15) Li, Y.; Gao, T.; Yang, Z.; Chen, C.; Luo, W.; Song, J.; Hitz, E.; Jia, C.; Zhou, Y.; Liu, B.; Yang, B.; Hu, L. 3D-Printed, All-in-One Evaporator for High-Efficiency Solar Steam Generation under 1 Sun Illumination. *Advanced Materials* **2017,** *29* (26), 1700981.

(16) Xu, W.; Hu, X.; Zhuang, S.; Wang, Y.; Li, X.; Zhou, L.; Zhu, S.; Zhu, J. Flexible and Salt Resistant Janus Absorbers by Electrospinning for Stable and Efficient Solar Desalination. *Advanced Energy Materials* **2018,** *8* (14), 1702884.

(17) Yang, Y.; Zhao, R.; Zhang, T.; Zhao, K.; Xiao, P.; Ma, Y.; Ajayan, P. M.; Shi, G.; Chen, Y. Graphene-Based Standalone Solar Energy Converter for Water Desalination and Purification. *ACS Nano* **2018,** *12* (1), 829-835.

(18) Xing, C.; Huang, D.; Chen, S.; Huang, Q.; Zhou, C.; Peng, Z.; Li, J.; Zhu, X.; Liu, Y.; Liu, Z.; Chen, H.; Zhao, J.; Li, J.; Liu, L.; Chen, F.; Fan, D.; Zhang, H. Engineering Lateral Heterojunction of Selenium-Coated Tellurium Nanomaterials toward Highly Efficient Solar Desalination. *Advanced Science* **2019**, 1900531.

(19) Zhu, L.; Ding, T.; Gao, M.; Peh, C. K. N.; Ho, G. W. Shape Conformal and Thermal Insulative Organic Solar Absorber Sponge for Photothermal Water Evaporation and Thermoelectric Power Generation. *Advanced Energy Materials* **2019,** 9, 1900250.

(20) Gao, M.; Peh, C. K.; Phan, H. T.; Zhu, L.; Ho, G. W. Solar Absorber Gel: Localized Macro-Nano Heat Channeling for Efficient Plasmonic Au Nanoflowers Photothermic Vaporization and Triboelectric Generation. *Advanced Energy Materials* **2018,** *8* (25), 1800711.

(21) Wang, X.; Liu, Q.; Wu, S.; Xu, B.; Xu, H. Multilayer Polypyrrole Nanosheets with Self-Organized Surface Structures for Flexible and Efficient Solar–Thermal Energy Conversion. *Advanced Materials* **2019,** *31* (19), 1807716.

(22) Zhu, L.; Gao, M.; Peh, C. K. N.; Wang, X.; Ho, G. W. Self-Contained Monolithic Carbon Sponges for Solar-Driven Interfacial Water Evaporation Distillation and Electricity Generation. *Advanced Energy Materials* **2018,** *8* (16), 1702149.

(23) Mu, P.; Bai, W.; Zhang, Z.; He, J.; Sun, H.; Zhu, Z.; Liang, W.; Li, A. Robust aerogels based on conjugated microporous polymer nanotubes with exceptional mechanical strength for efficient solar steam generation. *Journal of Materials Chemistry A* **2018,** *6* (37), 18183-18190.

(24) Yin, X.; Zhang, Y.; Guo, Q.; Cai, X.; Xiao, J.; Ding, Z.; Yang, J. Macroporous Double-Network Hydrogel for High-Efficiency Solar Steam Generation Under 1 sun Illumination. *ACS Applied Materials & Interfaces* **2018,** *10* (13), 10998-11007.

(25) Mu, P.; Zhang, Z.; Bai, W.; He, J.; Sun, H.; Zhu, Z.; Liang, W.; Li, A. Superwetting Monolithic Hollow-Carbon-Nanotubes Aerogels with Hierarchically Nanoporous Structure for Efficient Solar Steam Generation. *Advanced Energy Materials* **2019,** *9* (1), 1802158.

(26) Li, X.; Xu, W.; Tang, M.; Zhou, L.; Zhu, B.; Zhu, S.; Zhu, J. Graphene oxide-based efficient and scalable solar desalination under one sun with a confined 2D water path. *Proceedings of the National Academy of Sciences* **2016,** *113* (49), 13953.

(27) Xu, N.; Hu, X.; Xu, W.; Li, X.; Zhou, L.; Zhu, S.; Zhu, J. Mushrooms as Efficient Solar Steam-Generation Devices. *Advanced Materials* **2017,** *29* (28), 1606762.

(28) Ma, Q.; Yin, P.; Zhao, M.; Luo, Z.; Huang, Y.; He, Q.; Yu, Y.; Liu, Z.; Hu, Z.; Chen, B.; Zhang, H. MOF-Based Hierarchical Structures for Solar-Thermal Clean Water Production. *Advanced Materials* **2019,** *31* (17), 1808249.

(29) Li, C.; Jiang, D.; Huo, B.; Ding, M.; Huang, C.; Jia, D.; Li, H.; Liu, C.-Y.; Liu, J. Scalable and robust bilayer polymer foams for highly efficient and stable solar desalination. *Nano Energy* **2019,** *60*, 841-849.

(30) Zhang, P.; Li, J.; Lv, L.; Zhao, Y.; Qu, L. Vertically Aligned Graphene Sheets Membrane for Highly Efficient Solar Thermal Generation of Clean Water. *ACS Nano* **2017,** *11* (5), 5087-5093.

(31) Hu, X.; Xu, W.; Zhou, L.; Tan, Y.; Wang, Y.; Zhu, S.; Zhu, J. Tailoring Graphene Oxide-Based Aerogels for Efficient Solar Steam Generation under One Sun. *Advanced Materials* **2017,** *29* (5), 1604031.

(32) Yang, Y.; Zhao, H.; Yin, Z.; Zhao, J.; Yin, X.; Li, N.; Yin, D.; Li, Y.; Lei, B.; Du, Y.; Que, W. A general salt-resistant hydrophilic/hydrophobic nanoporous double layer design for efficient and stable solar water evaporation distillation. *Materials Horizons* **2018,** *5* (6), 1143-1150.

(33) Li, W.; Tekell, M. C.; Huang, Y.; Bertelsmann, K.; Lau, M.; Fan, D. Synergistic High-Rate Solar Steaming and Mercury Removal with MoS2/C @ Polyurethane Composite Sponges. *Advanced Energy Materials* **2018,** *8* (32), 1802108.

(34) Xu, W.; Xing, Y.; Liu, J.; Wu, H.; Cui, Y.; Li, D.; Guo, D.; Li, C.; Liu, A.; Bai, H. Efficient Water Transport and Solar Steam Generation via Radially, Hierarchically Structured Aerogels. *ACS Nano* **2019,** *13* (7), 7930-7938.

(35) Cui, L.; Zhang, P.; Xiao, Y.; Liang, Y.; Liang, H.; Cheng, Z.; Qu, L. High Rate Production of Clean Water Based on the Combined Photo-Electro-Thermal Effect of Graphene Architecture. *Advanced Materials* **2018,** *30* (22), 1706805.

(36) Sun, Z.; Wang, J.; Wu, Q.; Wang, Z.; Wang, Z.; Sun, J.; Liu, C.-J. Plasmon Based Double-Layer Hydrogel Device for a Highly Efficient Solar Vapor Generation. *Advanced Functional Materials* **2019,** *0* (0), 1901312.

(37) Zhou, X.; Zhao, F.; Guo, Y.; Rosenberger, B.; Yu, G. Architecting highly hydratable polymer networks to tune the water state for solar water purification. *Science Advances* **2019,** *5* (6), eaaw5484.
